# Genome Wide Analysis of Fertility and Production Traits in Italian Holstein Cattle 

**DOI:** 10.1371/journal.pone.0080219

**Published:** 2013-11-12

**Authors:** Giulietta Minozzi, Ezequiel L. Nicolazzi, Alessandra Stella, Stefano Biffani, Riccardo Negrini, Barbara Lazzari, Paolo Ajmone-Marsan, John L . Williams

**Affiliations:** 1 Parco Tecnologico Padano, Lodi, Italy; 2 Institute of Zootechnics, Università Cattolica del Sacro Cuore, Piacenza, Italy; 3 Istituto di Biologia e Biotecnologia Agraria, Consiglio Nazionale delle Ricerche, Lodi, Italy; 4 Associazione Nazionale Allevatori Frisona Italiana, Cremona, Italy; 5 Associazione Italiana Allevatori, Rome, Italy; 6 Nutrigenomics Research Center, Università Cattolica del Sacro Cuore, Piacenza, Italy; The University of Chicago, United States of America

## Abstract

A genome wide scan was performed on a total of 2093 Italian Holstein proven bulls genotyped with 50K single nucleotide polymorphisms (SNPs), with the objective of identifying loci associated with fertility related traits and to test their effects on milk production traits. The analysis was carried out using estimated breeding values for the aggregate fertility index and for each trait contributing to the index: angularity, calving interval, non-return rate at 56 days, days to first service, and 305 day first parity lactation. In addition, two production traits not included in the aggregate fertility index were analysed: fat yield and protein yield. Analyses were carried out using all SNPs treated separately, further the most significant marker on BTA14 associated to milk quality located in the DGAT1 region was treated as fixed effect. Genome wide association analysis identified 61 significant SNPs and 75 significant marker-trait associations. Eight additional SNP associations were detected when SNP located near *DGAT1* was included as a fixed effect. As there were no obvious common SNPs between the traits analyzed independently in this study, a network analysis was carried out to identify unforeseen relationships that may link production and fertility traits.

## Introduction

Herd fertility is important for the economic sustainability of dairy cattle farming. However, the fertility of Holstein cattle is steadily decreasing [1], and is becoming one of the main causes of culling and replacement of cows in dairy herds worldwide. Fertility is affected by several male and female characteristics, many of which are difficult and expensive to measure accurately. Dairy cattle selection programs are therefore often focused on fertility phenotypes that have low heritability, some of which are associated with the high level of milk production. These phenotypes have been used to create fertility selection indexes that may include production traits, typically milk yield, in addition to measures of fertility [2]. 

Linkage mapping studies have localised genetic loci putatively associated with reproductive and production traits in dairy cattle [3,4] and their relative genetic and phenotypic correlation have been tested [5,6,7]. In addition candidate genes with effects on fertility traits have been described, including Plasma Protein-A2 (*APP2*) on chromosome 16 [8] and calpastatin (*CAST*), which are expressed in reproductive organs [9]. Further, studies of Single Nucleotide Polymorphisms (SNPs) within genes showing differential expression in the bovine endometrium during luteal and ovulatory phases have identified loci with genetic effects on fertility and production traits [10]. 

Genomic estimation of breeding values of bulls using SNPs and phenotypic data collected on their progeny are being used in selection programmes. The data used for the genomic estimates can also be used for genome wide association studies to identify genetic loci with major effects on fertility and other traits. Recently, Genome Wide Association Studies (GWAS) of female fertility traits have been carried out using 50K SNP genotypes in Holstein-Friesian, Finnish Ayrshire, and Jersey breeds [6,11,12,13]. Significant marker-trait associations have been described on several chromosomes, however only a few chromosomal regions (e.g BTA13 and BTA18) were consistently identified in these studies with the same SNPs associated with fertility traits across investigations, and hence the chromosomal regions implicated were not exactly the same. These differences may be due to genetic heterogeneity among the populations studied, that the trait measurements were not the same, differences in sample size, power of the analyses or environmental influences (eg. experimental/commercial herds).

The GWAS reported here used data from Italian Holstein proven bulls genotyped with 50K SNPs, with the objective of identifying loci associated with fertility related traits and to test their effects on milk production traits. The analysis was carried out using EBVs for the aggregate fertility index traditionally used in selection for Italian Holstein cows [2] and for each trait contributing to the index: angularity (ANG), calving interval (CI), non-return rate at 56 days (NR56), days to first service (DFS), and 305 day first parity lactation (MILK). In addition, two production traits not included in the aggregate fertility index were analysed: fat yield (FAT) and protein yield (PROT). Further, the most significant marker on BTA14 associated to milk quality traits located in the DGAT1 region, was treated as fixed effect to discover other significant markers eventually masked by the large effect of the SNP on fat content and correlated traits.

## Materials and Methods

### Animals

A total of 2139 Holstein bulls progeny tested in Italy were chosen based on the: i) availability of biological material or DNA samples; ii) reliability of at least 75% for the Production, Functionality, Type (PFT) national selection index. PFT takes into account both production and functional traits in the proportion 49% : 51% ; iii) largest number of families and balanced number of members per family, to maximize the representativeness of the population. In detail the dataset contained 204 son-father pairs. Semen samples (straws) were provided by Italian certified artificial insemination centers (EU Directive 88/407/CEE), thus ethics committee approval for this study is not required. 

### Genotyping and quality control

Bulls were genotyped with the BovineSNP50 BeadChip (Illumina, San Diego, CA). Genotypes were quality controlled and markers excluded for missing data (≥ 2.5%), minor allele frequency (≤ 5%), mendelian errors identified for father-son pairs (≥ 2.5%), and deviation from Hardy-Weinberg equilibrium (HWE; p ≤ 0.001, Bonferroni corrected). Bulls with more than 5% missing data were also removed. A mendelian transmission check was performed on father-son pairs. Sons of father-son pairs with unexpectedly high (≥100) mendelian errors were excluded. 

Classical Multi Dimension Scaling (MDS) was performed within the R statistical environment using the GenABEL package [14] to explore population structure and verify the genetic homogeneity of the dataset prior to carrying out the GWAS. 

### Phenotypes

Official EBVs for eight fertility and production traits were provided by the Italian Holstein breeder association (ANAFI; table 1). Fertility traits included the aggregate fertility index (FRT) used to select for fertility in Italian Holstein cows and the five traits from which it is compose: angularity (ANG), calving interval (CI), non return rate at 56 days (NR56), days to first service (DFS), and first parity mature equivalent milk yield at 305 days (MILK;[2]). EBVs for the five component traits were obtained using a multivariate animal model with all traits fitted simultaneously. This multi-trait approach allowed (co)variances between traits to be estimated, which were then used to calculate the aggregate index. In addition, two production traits were analysed, fat (FAT) and protein (PROT) yield. EBVs for FAT and PROT were obtained from the official ANAFI evaluation which is based on a multiple trait, multiple lactation Random Regression Test Day Model (RRTDM), as described in Muir et al. (2007)[15]. Heritability for the fertility traits analysed were 0.17 for ANG, 0.07 for CI, 0.03 for NR56, 0.06 for DFS and 0.026 for the FRT Fertility Index [2]. 

**Table 1:** Mean, standard deviation and number of observations of deregressed EBVs of the traits investigated.

Trait	N	Mean	Sd
Angularity (ANG)	1994	0.06	1.53
Calving Interval (CI)	1994	3.87	13.79
Non return rate (NR56)	1994	-0.01	0.03
Days to 1st service (DFS)	1994	1.41	6.41
Fertility Index (FRT)	1994	101.07	5.53
Milk yield (MILK)	1994	97.95	727.12
Fat yield (FAT)	1994	2.27	25.43
Protein yield (PROT)	1994	4.52	22.47

The number of animals included in the GWAS, and the mean and standard deviations of EBVs for all the traits analyzed are shown in Table 1.

### Analysis

Genome-wide association analysis was performed with the GenABEL package in R using a three step GRAMMAR-GC (Genome wide Association using Mixed Model and Regression - Genomic Control) approach [16,17], using the genomic relationship matrix estimated from SNP data instead of pedigrees. First an additive polygenic model was used to obtain individual environmental residuals using the polygenic function of the GenABEL library to disentangle the cryptic population structure caused by the presence of closely related animals in the sample set [16]. To account for relatedness, the variance/covariance matrix was estimated from the genomic kinship matrix, as pedigree information was not available. The relationship matrix used in the analysis was estimated using genomic data with the ‘‘ibs’’ (option weight= ‘‘freq’’) function of GenABEL. Secondly, association was tested using a simple least squares method using an additive model on the residuals, corrected for cryptic relatedness, familiar correlation, and independent of pedigree structure. Thirdly, the Genomic Control (GC) approach was used to correct for conservativeness of the GRAMMAR test, based on the estimation of the lambda factor, which is the median of all genome-wide observed test statistics divided by the expected median of the test statistic under the null hypothesis of no association, assuming that the number of true associations is very small compared to the number of tests that are actually performed.Uncorrected p-values of ≤ 5 x e-05were considered as significant associations (Wellcome Trust Case Control Consortium 2007). 

After testing all SNPs together, a second GWAS analysis was carried out including the SNP ARS-BFGL-NGS-4939 on BTA14 at position 1801116 bp, as a fixed effect, to account for the known large effect of the nearby *DGAT1* gene (diacylglycerol O-acyltransferase 1) on production traits. The K232A mutation in the *DGAT1* gene that affects milk traits is known to segregate in the Italian Holstein population (*Fontanesi et al., 2013 unpublished data*). 

### Network analysis

Genes located within 1 Mbp upstream and downstream of significant SNPs were retrieved from the Ensembl database. Location and gene annotation were based on the UMD 3.1 genome assembly and on Ensembl release 65 (http://www.ensembl.org). Canonical transcripts were blasted against the Human RefSeq and the Uniprot databases to search annotations for unannotated cow genes by retrieving homologous human transcript IDs. The Bovine Ensemble protein IDs were analyzed using Ingenuity Pathways Analysis software (IPA, Ingenuity® Systems, www.ingenuity.com). Each identifier was then mapped to its corresponding gene object in the Ingenuity Pathways Knowledge Base. Finally, networks of genes were generated on the basis of their connectivity. IPA assignes a score to each network (based on *P* value) representing the likelihood that the focus genes within the network are found therein by random chance. A high number of focus genes within a dataset leads to a higher network score. 

## Results

### Genotyping and quality control

Following quality control, markers with excessive missing data (2102 SNPs), low minor allele frequency (10643 SNPs), high mendelian error rate (177 SNPs) and because they were out of HWE (79 SNPs) were excluded. Markers without position information and on sex chromosomes were also removed (2,465 SNPs). Thirty-eight samples were removed because of low genotyping rate, and 8 because of excessive mendelian errors. No outlier was identified by Classical Multi Dimension Scaling (MDS, data not shown). The working dataset contained 2,093 samples and 38,535 SNPs. In each GWAS run, samples missing phenotype data for the specific trait were eliminated. 

### Results of the association analysis

**Figure 1:**
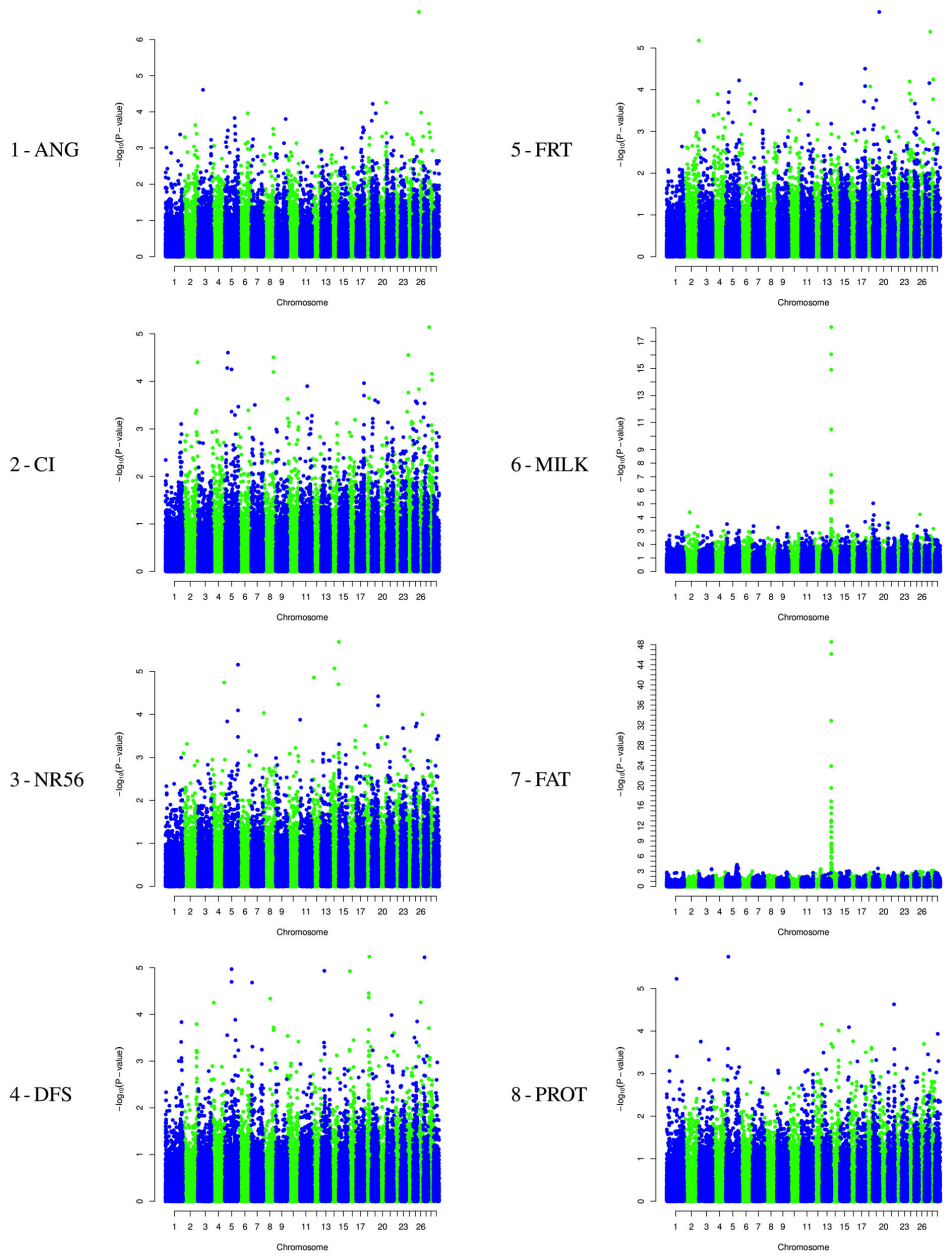
Manhattan plot displaying the results of the Genome-wide scan with respect to their genomic position.

GWAS identified 61 significant SNPs and 75 significant marker-trait associations with the single marker model. The genome-wide Manhattan Plots and details on these marker-trait associations are shown in Figure 1 and Table 2. Eight additional SNP associations were detected when SNP ARS-BFGL-NGS-4939, located near *DGAT1* was included as a fixed effect. Among the significant 69 SNPs, 58 were associated with a single trait, 8 with two traits and 3 with three traits. The three SNPs associated with three traits were all in the proximal 1Mbp of BTA14 (ARS-BFGL-NGS-57820; ARS-BFGL-NGS-4939 and UA-IFASA-6878), associated with MILK, FAT and PROT. Five of the 8 SNPs associated with two traits mapped in the same 1Mbp BTA14 region (Hapmap30383-BTC-005848; Hapmap52798-ss46526455; ARS-BFGL-NGS-107379; Hapmap25384-BTC-001997 and Hapmap24715-BTC-001973), all associated with MILK and FAT. The 3 other SNPs were associated with CI and FRT, on the distal region of BTA2 (ARS-BFGL-NGS-102467 at 136,188,090bp) and on BTA28 (BTB-00974967 at 8,033,988bp) and with ANG and MILK on BTA26 (ARS-BFGL-NGS-4670 at 9,550,462bp). 

**Table 2:** List of the top SNP identified for each trait.

Angularity (ANG)						
SNP name	BTA	Position (bp)	N	effB	Pc1df	MAF
Hapmap43908-BTA-67758 ^ab^	3	44959256	1993	-0,04	2.43 e-05	0,16
ARS-BFGL-NGS-4670 ^ab^	26	9550462	1992	-0,07	1.74 e-07	0,07
Calving Interval (CI)						
SNP name	BTA	Position (bp)	N	effB	Pc1df	MAF
ARS-BFGL-NGS-102467 ^ab^	2	136188090	1992	0,28	3.98 e-05	0,25
BTA-72978-no-rs ^ab^	5	25699752	1993	-0,54	2.46 e-05	0,05
ARS-BFGL-NGS-8769 ^ab^	8	91642318	1994	-0,52	3.10 e-05	0,06
Hapmap59517-rs29027550 ^ab^	24	20886644	1993	-0,28	2.77 e-05	0,25
BTB-00974967 ^ab^	28	8033988	1985	-0,34	7.16 e-06	0,18
Non return rate (NR56)						
SNP name	BTA	Position (bp)	N	effB	Pc1df	MAF
ARS-BFGL-NGS-73498 ^ab^	4	115946071	1993	-0,0007	1.81 e-05	0,17
ARS-BFGL-NGS-7979 ^ab^	5	116553827	1994	0,0006	6.91 e-06	0,44
ARS-BFGL-NGS-107895 ^ab^	12	20720170	1994	0,0012	1.36 e-05	0,06
UA-IFASA-8273 ^ab^	14	44765662	1994	-0,0013	8.38 e-06	0,05
BTA-120049-no-rs ^ab^	14	82745092	1979	0,0006	1.95 e-05	0,28
ARS-BFGL-NGS-118371 ^ab^	14	84145486	1993	0,001	2.04 e-06	0,09
ARS-BFGL-NGS-39839 ^ab^	19	63261405	1993	0,0006	3.73 e-05	0,29
Days to first Service (DFS)						
SNP name	BTA	Position (bp)	N	effB	Pc1df	MAF
ARS-BFGL-NGS-110018 ^ab^	5	60642347	1991	0,24	1.06 e-05	0,19
Hapmap60306-rs29023088 ^ab^	5	60665676	1994	0,25	2.00 e-05	0,15
ARS-BFGL-NGS-25179 ^ab^	7	7822111	1992	-0,27	2.06 e-05	0,12
BTB-00351490 ^ab^	8	61752353	1980	-0,37	4.56 e-05	0,06
BTA-32199-no-rs ^ab^	13	30530185	1994	-0,21	1.16 e-05	0,27
BTA-40382-no-rs ^ab^	16	22101902	1994	0,21	1.18 e-05	0,27
Hapmap54118-rs29014623 ^ab^	18	42435476	1994	0,18	4.33 e-05	0,33
ARS-BFGL-NGS-113068 ^ab^	18	43165294	1990	0,18	3.52 e-05	0,35
ARS-BFGL-BAC-35026 ^ab^	18	48150900	1991	0,26	5.80 e-06	0,16
BTB-00951196 ^ab^	27	10512202	1950	0,25	6.01 e-06	0,19
Aggregate Fertility Index (FRT)						
SNP name	BTA	Position (bp)	N	effB	Pc1df	MAF
ARS-BFGL-NGS-102467 ^ab^	2	136188090	1992	-0,11	6.60 e-06	0,25
ARS-BFGL-NGS-74168 ^ab^	17	73488482	1994	0,14	3.09 e-05	0,09
ARS-BFGL-NGS-72483 ^ab^	19	63501603	1994	0,21	1.37 e-06	0,06
BTB-00974967 ^ab^	28	34722075	1985	0,12	4.02 e-06	0,18
UA-IFASA-7767 ^b^	28	51200785	1993	0,08	4.64 e-05	0,47
Milk Yield (MILK)						
SNP name	BTA	Position (bp)	N	effB	Pc1df	MAF
BTA-47612-no-rs ^ab^	2	51162276	1994	-20,5	4.33 e-05	0,05
BTB-00098202 ^ab^	2	51200785	1994	-20,5	4.33 e-05	0,05
Hapmap30383-BTC-005848 ^a^	14	1489496	1993	-15,68	3.11 e-11	0,33
ARS-BFGL-NGS-57820 ^a^	14	1651311	1993	-23,28	9.15 e-17	0,2
ARS-BFGL-NGS-4939 ^a^	14	1801116	1994	-25	8.60 e-19	0,19
Hapmap52798-ss46526455 ^a^	14	1923292	1990	-11,94	7.06 e-08	0,48
ARS-BFGL-NGS-107379 ^a^	14	2054457	1990	-20,66	1.21 e-15	0,25
Hapmap25384-BTC-001997 ^a^	14	2217163	1993	-10,94	1.43 e-06	0,43
Hapmap24715-BTC-001973 ^a^	14	2239085	1994	-11,05	1.01 e-06	0,43
UA-IFASA-6878 ^a^	14	2002873	1993	-10,24	5.47 e-06	0,43
Hapmap25486-BTC-072553 ^a^	14	1292993	1985	10,76	7.91 e-06	0,35
UA-IFASA-8401 ^ab^	19	7461446	1993	10,11	8.87 e-06	0,45
ARS-BFGL-NGS-37177 ^b^	19	13154786	1989	13,14	3.34 e-05	0,18
ARS-BFGL-NGS-4670 ^b^	26	9550462	1992	-20,38	9.23 e-06	0,07
Fat yield (FAT)						
SNP name	BTA	Position (bp)	N	effB	Pc1df	MAF
Hapmap23365-BTA-156277 ^b^	5	91118692	1967	-0,52	2.33 e-05	0,24
Hapmap53294-rs29016908 ^b^	5	94645698	1994	0,56	5.89 e-06	0,23
ARS-BFGL-NGS-116897 ^b^	5	95743746	1993	-0,47	1.19 e-05	0,39
ARS-BFGL-NGS-12289 ^ab^	5	97223172	1994	-0,78	4.78 e-05	0,11
ARS-BFGL-NGS-17216 ^b^	5	107984945	1994	0,6	3.67 e-05	0,16
Hapmap30381-BTC-005750 ^a^	14	1463676	1994	-0,52	3.88 e-05	0,36
Hapmap30383-BTC-005848 ^a^	14	1489496	1993	0,93	1.75 e-13	0,33
ARS-BFGL-NGS-57820 ^a^	14	1651311	1993	2,16	0	0,2
ARS-BFGL-NGS-34135 ^a^	14	1675278	1993	1,07	1.31 e-17	0,4
ARS-BFGL-NGS-94706 ^a^	14	1696470	1992	1,02	1.99 e-16	0,41
ARS-BFGL-NGS-4939 ^a^	14	1801116	1994	2,24	0	0,19
Hapmap52798-ss46526455 ^a^	14	1923292	1990	1,1	3.00 e-20	0,48
ARS-BFGL-NGS-107379 ^a^	14	2054457	1990	1,67	0	0,25
ARS-BFGL-NGS-18365 ^a^	14	2117455	1990	-1,02	3.11 e-15	0,34
Hapmap30922-BTC-002021 ^a^	14	2138926	1994	-0,89	1.52 e-11	0,31
UA-IFASA-8997 ^a^	14	2194228	1994	-0,6	1.91 e-05	0,24
Hapmap25384-BTC-001997 ^a^	14	2217163	1993	0,71	5.13 e-09	0,43
Hapmap24715-BTC-001973 ^a^	14	2239085	1994	0,68	1.56 e-08	0,43
BTA-35941-no-rs ^a^	14	2276443	1994	0,94	7.69 e-14	0,37
ARS-BFGL-NGS-26520 ^a^	14	2386688	1994	-0,61	1.26 e-06	0,39
UA-IFASA-6878 ^a^	14	2002873	1993	1,23	0	0,43
ARS-BFGL-NGS-22866 ^a^	14	2826632	1994	0,51	2.44 e-05	0,48
ARS-BFGL-NGS-103064 ^a^	14	2754909	1994	0,49	4.45 e-05	0,47
Hapmap30646-BTC-002054 ^a^	14	2553525	1994	-0,59	1.05 e-06	0,45
Hapmap30086-BTC-002066 ^a^	14	2524432	1992	0,78	1.80 e-10	0,48
Hapmap30374-BTC-002159 ^a^	14	2468020	1993	0,89	1.56 e-12	0,38
ARS-BFGL-NGS-74378 ^a^	14	3640788	1994	0,76	5.09 e-08	0,24
ARS-BFGL-BAC-1511 ^a^	14	3765019	1994	-0,96	5.84 e-08	0,13
UA-IFASA-9288 ^a^	14	3956956	1994	0,79	2.55 e-08	0,23
ARS-BFGL-NGS-56327 ^a^	14	4336714	1994	0,62	2.79 e-06	0,29
ARS-BFGL-NGS-100480 ^a^	14	4364952	1994	0,77	2.67 e-09	0,34
UA-IFASA-5306 ^a^	14	4468478	1994	0,74	1.72 e-07	0,23
ARS-BFGL-NGS-39328 ^b^	19	51326750	1993	0,45	3.84 e-05	0,35
Protein Yield (PROT)						
SNP name	BTA	Position (bp)	N	effB	Pc1df	MAF
Hapmap27148-BTA-124406 ^ab^	1	89941301	1994	0,43	1.51 e-05	0,08
BTA-27242-no-rs ^ab^	5	19931600	1994	-0,51	4.03 e-06	0,05
ARS-BFGL-NGS-57820 ^a^	14	1651311	1993	0,31	3.95 e-06	0,2
ARS-BFGL-NGS-4939 ^a^	14	1801116	1994	0,27	4.00 e-05	0,19
UA-IFASA-6878 ^a^	14	2002873	1993	0,23	9.71 e-06	0,44
ARS-BFGL-NGS-44370 ^a^	15	84778401	1993	-0,41	4.30 e-05	0,08
ARS-BFGL-NGS-110401 ^ab^	21	62769411	1975	0,23	3.11 e-05	0,34

SNP name= snp name as in the bovine 50K SNP Chip dataBTA= *Bos Taurus* Autosome Position= position (bp) on UMD 3.1N= number of animals tested for the specific markerPc1df= p-values adjusted for Genomic ControleffB = Effect of the minor allele (B allele).MAF=minor allele frequency

Note: SNPs with a superscript “a” are detected by single marker analysis only, SNPs with a superscript “b” are detected by including the SNP close to the DAGT1 gene as fixed effect in the model only, SNPs with a superscript “ab” are detected by both models. Markers are ordered according to BTA and position within traits.

Four regions contained more than one significant SNP. The proximal region of BTA14 between 1.2 and 2.8Mbp contained 21 SNPs (Tab. 2), associated with either two (MILK and FAT) or to all three of the production traits investigated; a region on BTA2, at 51.2Mbp contained 2 SNPs (BTA-47612-no-rs and BTB-00098202) associated with MILK; a region on BTA 5, at 60.6Mbp had 2 SNPs) (ARS-BFGL-NGS-110018 and Hapmap60306-rs29023088) associated with DFS and a region on BTA19, at 63.4Mbp, where two markers (ARS-BFGL-NGS-39839 and ARS-BFGL-NGS-72483) were respectively associated with NR56 and FRT.

The number of significant associations per trait ranged from a minimum of 2 for ANG to a maximum of 33 for FAT (Table 2). The number of associated SNPs was higher for production compared with fertility traits, but comparable when closely linked SNPs were considered as marking a single region. In total, 36 SNPs and 26 regions were associated with the 5 traits (ANG, CI, NR56, DFS and MILK) included the aggregated FRT index. Conversely FRT was significantly associated with 5 regions, 2 of which were in common, associated with CI (on BTA2 and BTA28) and which were not 3 detected by the single trait analysis (on BTA17, BTA19 and BTA28; Table 2).

Significant associations were detected on 20 of the 29 bovine autosomes (Table 3). The greatest number of associations was found on BTA14 (31 SNPs for four traits: FAT, MILK, PROT and NR56), followed by BTA5 (10 SNPs for five traits: CI, DFS, FAT, NR56 and PROT). BTA19 had 5 significant SNPs for four traits (FAT, FRT, MILK and NR56). BTA2 and BTA18 had 3 significant SNPs, BTA8, BTA26 and BTA28 2 SNPs and the other twelve chromosomes (BTA1, 3, 4, 7, 12, 13, 15, 16 17, 21, 24 and 27) had a single SNP for the single trait.

**Table 3:** Allele substitution effect (effB) and probability of association between marker BTA-27242-no-rs on BTA5 and traits investigated. The number of observations per trait is also indicated.

Trait	n	effB	P-value (Pc1df)
Angularity (ANG)	1994	-0,05	4.97 e-04
Calving Interval (CI)	1994	-0,4982	5.21 e-05
Non return rate (NR56)	1994	0,0002	3.35 e-01
Days to 1st service (DFS)	1994	-0,3221	2.76 e-04
Fertility Index (FRT)	1994	0,1561	1.99 e-04
Milk yield (MILK)	1994	-14,896	1.29 e-03
Fat yield (FAT)	1994	-0,739	2.96 e-03
Protein yield (PROT)	1994	-0,5027	1.79 e-06

### Results of the network analysis

**Figure 2:**
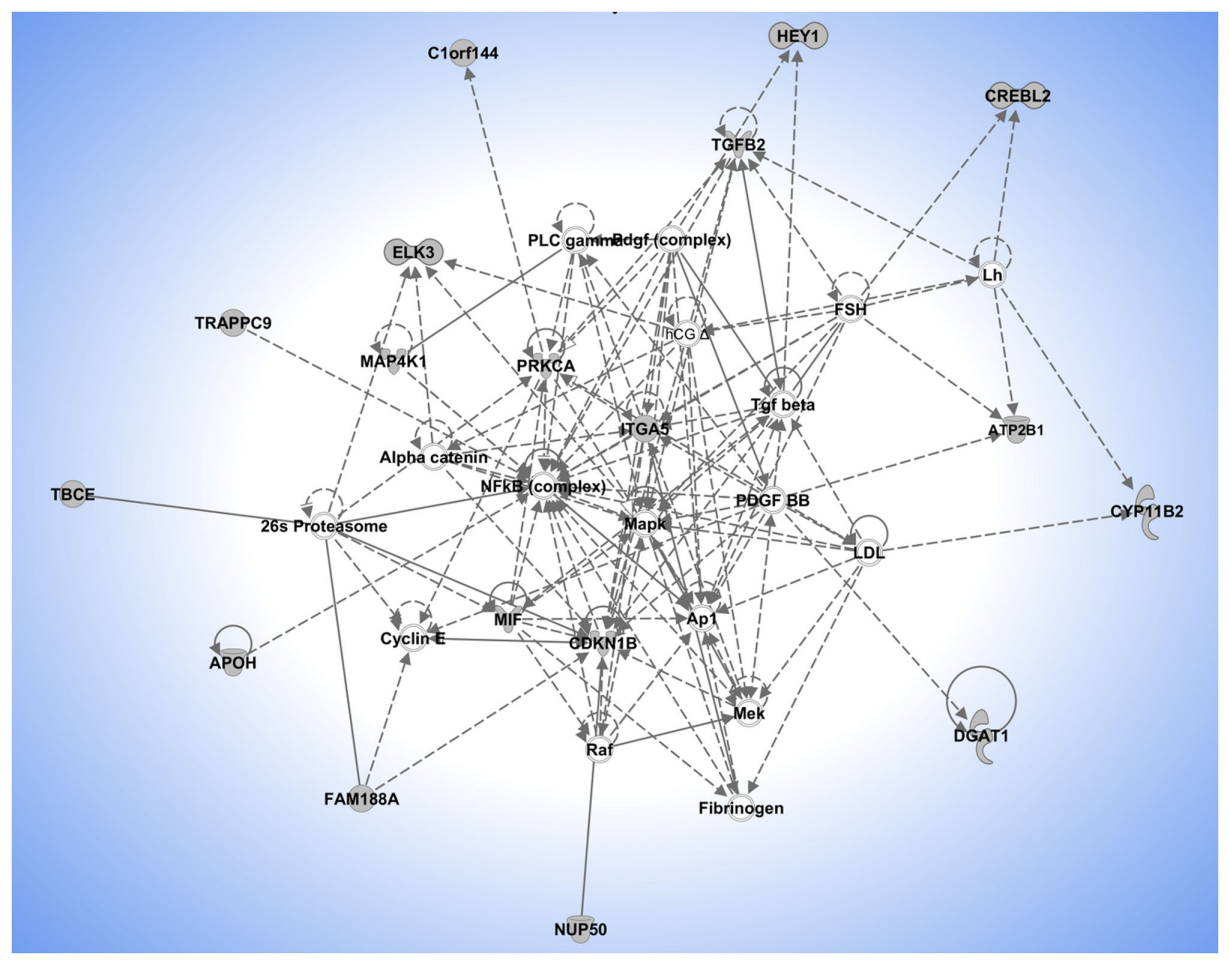
Network 2 generated by the Ingenuity Pathway analysis software. Genes identified under the main peaks of the genome scan are shown in grey, while the symbols without color are the genes added by Ingenuity Pathway Analysis Software to produce the network (connecting genes). Lines in the network indicate direct interaction, while dotted lines indicate indirect interactions. Nodes are displayed using various shapes that represent the functional class of the gene product. The composite score of the network is 34, that represents the negative log of the p-value for the likelihood that the molecules would be found together by chance. A higher score indicates greater probability that the molecules in the network are interconnected.

In total 118 genes located within 1Mbp of significant SNPs were used in the IPA network analysis. Five main networks were identified. The first had an IPA score of 37 and includes genes involved in Cell Death, Cellular Development and Cell Signaling. The second network was comprised of genes involved in Gene Expression, DNA Replication, Recombination, Repair, and Lipid Metabolism, and had a score of 34 (Figure 2). Three other networks were identified, with scores lower or equal to 30, containing genes involved in Amino Acid Metabolism, Molecular Transport, Small Molecule Biochemistry (IPA score = 30); Cell Cycle, Drug Metabolism, Gene Expression (IPA score = 24); Cell Death, Cell Morphology, Cellular Development (IPA score = 18).

## Discussion

The genome wide association studies described here detected in total 69 significant markers and 83 marker/trait associations with individual fertility traits, the aggregated fertility index and the three production traits (P ≤ 5 x e-05). A larger number of significant associations were identified for production (54) than for fertility traits (29). In addition, the associations with production traits showed higher significance levels and lower false discovery rates (Table 3). This may be expected from their higher heritability and EBV reliability of the production traits compared with single the fertility traits and the composite fertility index (Table 1). Fertility traits are highly affected by environmental conditions, which are difficult to measure. Additionally these traits are not the biological processes that determine fertility, but are correlated measures which are also affected by management decisions. These factors contribute to decrease the power of detecting associations with the markers. Among the significant SNPs, only 21 showed highly significant associations (P ≤ 5 x e-07), and these were with three traits, two production traits that are precise measurements (MILK and FAT) and the third trait which has subjective, but well defined measurements (ANG). These significant SNPs were on two chromosomes, BTA14 and BTA26.

Considering significant markers closer than two Mbp as defining a same QTL region, 22 regions were associated with fertility traits and 14 with production traits. In general, regions associated with the fertility traits were marked by fewer SNPs than the production traits. This is due to the weaker association of SNPs associated to fertility traits, compared to production traits, and hence for fertility traits often only a single marker in a QTL peak exceeded the significance threshold.

### The effect of DGAT1

When the SNP close to *DGAT1* on BTA14 was included as a fixed effect, only 43 marker-trait associations were significant, which were not completely overlapping with the 69 identified when SNP close to *DGAT1* was included in the analysis. These 43 significant SNP were located at 20 independent chromosome locations.. Interestingly, some were common with the ones identified when the *DGAT1* SNP was not included in the model, while 8 SNPs became significant only when considering the effect of the marker nearby *DGAT1* in the model. These new significant associations were found on chromosomes 5, 19, 26 and 28 and were associated with FAT, MILK and FRT. This suggests that in populations of limited size undergoing strong selection, major QTL for a trait (DGAT1 in this case) may mask loci with a lower effect on the same or correlated traits (FAT and MILK in this study). There is extensive evidence that DGAT1 gene influences production traits [18,19], and has an impact on the MILK trait considered here. MILK is among the five traits used to calculate FRT [2], which was the only trait showing an increased p-value when accounting for the effect of DGAT1. Therefore, it is likely that the apparent effect of the chromosomal locus containing DGAT1 on fertility is through the inclusion of MILK in the FRT. The remaining 35 significant associations were always observed in our results when the DGAT1 associated SNP was included or excluded from the analysis. 

### GWAS of fertility

A total of 38 SNPs were significantly associated with traits included in FRT index (ANG=2; CI=5; NR56=7; DFS=10; MILK=14). Only 3 of these SNPs were significantly associated with the FRT composite index. The three SNPs in common were on BTA2 and BTA28 for CI and on BTA19 for NR56, which are the two traits with highest relative weight in the index (CI=51%; NR56=17%). Low SNP effects, epistatic interactions, and/or the relative low weight in FRT of the other individual traits may have reduced the chance of detecting the association of the other 35 markers with the composite index. Interestingly, FRT was associated with two regions not detected for the individual traits, on BTA17 and BTA28. Pleiotropy or epistasis may explain this finding. No QTL was found in common between the individual fertility traits included in the FRT index (Table 2). 

A recent study based on the Finnish Ayrshire breed identified SNPs on chromosomes 1, 2, 4, 12, 13, 20, 24 and 27, associated with non-return rate, time from first to last insemination, number of inseminations and time from calving to first insemination [13]. Some of these traits are similar to those analyzed in the present study, however, only one chromosomal region is in common. In the present study a QTL on BTA27 at 10.5Mbp was associated with DFS while Shulman et al. detected a QTL for non return rate for heifers at 9.3Mbp on UMD 3.1 (quoted as 11.2Mbp in the original publication based on the Btau 4.0 assembly). However, the distance between the two QTL and the different nature of the trait definitions gives little confidence that the same locus has been identified.

Other recently published genome wide studies in dairy cattle have reported markers associated with fertility traits [5,12,11]. In all cases regions reported are either distant from those identified in the present study or supported by very large confidence intervals. For example, the significant association with NR56 reported here on BTA12 at position 20.1Mbp lies within the confidence interval of a QTL for the same trait reported by Hoglund et al. (2008) [5]. The Hoglund et al.. QTL confidence interval is so large (11Mbp - 47Mbp) that comparison is difficult. In the same region of BTA12, a QTL for first to last insemination in days was found in Finnish Ayrshire at 24.3 Mbp (Schulman et al., 2008). In this case the trait definition is consistent with that of the present study, however, the distance is such that the loci detected are unlikely to be the same. 

### Functional candidate genes for fertility

A literature review based on recently published association or expression studies (Table S2) identified 559 candidate genes associated with fertility traits or differentially expressed in high and low fertility individuals in different mammalian species (Table S1). Among these candidate genes, 6 mapped within 2Mbp of significant markers identified in the present study.

The carnitine palmitoyl transferase 1B gene (*CPT 1B*) maps on BTA5 approximately 2Mbp from ARS-BFGL-NGS-7979 which was associated with NR56. CPT *1B* is involved in lipid metabolism and transport, and has been shown to be up-regulated in dairy cattle endometrium at day 17 of pregnancy [20]. At implantation the uterus is in a highly active metabolic state and variations in this gene may affect early embryonic losses, and hence return rates. 

The Insulin-like growth factor 1 gene (*IGF1*) is also located on BTA5, close to two SNPs associated with DFS (ARS BFGL-NGS-25179 and Hapmap60306-rs29023088, see Table 2). An association was found between the SNP SnaBI in *IGF1* and interval from calving to commencement of luteal activity postpartum (CLA) in a study of fertility, milk production and body condition in Holstein-Friesian dairy cows [21].

The anti-müllerian hormone receptor type II gene (*AMHR2*) is located on BTA5 under the peak centered on BTA-72978-no-rs, associated with CI (Table 2). Several TGF-β superfamily members and their receptors are involved in folliculogenesis, including *AMHR2* [22]. Anti-müllerian hormone is an early follicle growth inhibitor that seems to be the only ligand of AMHR2 [22] . *AMHR2* mRNA levels in granulosa cells of small follicles have been found to be elevated in cases of polycystic ovary syndrome in humans [23]. This dys-regulation of growth factors potentially alters the intra-follicular environment impairing the maturation of the oocyte and hence will influence the calving interval [22].

The transcription growth factor b2 (*TGFB2*) and the apolipoprotein H (*APOH*) genes are under the peaks identified by BTA-40382-no-rs on BTA 16 and ARS-BFGL-NGS-39839 on BTA 19, respectively associated to DFS and NR56 (Table 2). Both TGFB2 and APOH are involved in the process of the follicular development as they interact with the reproductive hormones LH and FSH and so could be considered as candidate genes affecting fertility [24]. 

The immunoglobulin lambda-like polypeptide 1 precursor (*IGLL1*) is located near to ARS-BFGL-NGS-74168 on BTA17 which was associated with FRT. The *IGLL1* gene has been found to be up-regulated during the peripartum period in the endometrium in lactating vs. non-lactating dairy cattle [20]. This suggests that this gene may play a role in energy balance, and influence production and fertility traits.

Only one SNP was associated with more than one trait: ARS-BFGL-NGS-4670 on BTA26 was associated with MILK and ANG. It is not known if this SNP marks the same QTL which affects both of these traits, or if there is a fortuitous co-location of two different QTL. There are two genes located within 1 Mbp of this SNP. Phosphatase and tensin homolog (*PTEN*), which is involved in regulating the cell cycle and in endometrial cancer in humans [25] and Lipase family protein J (*LIPJ*), a gene with testis specific expression that belongs to the family of mammalian acid lipases and superfamily of AB hydrolases that catalyse the hydrolysis of triglycerides in the body. Neither of these genes has an obvious role in the quantitative traits associated with this chromosomal region. 

### Analysis of interaction between fertility and production traits

The relationship between fertility and production was investigated using two strategies: i) SNPs significant for a trait were examined to identify if they may be associated with another trait which fell slightly below the p-value threshold set for significance and ii) a network analysis was performed taking into account all genes identified under the main peaks, to identify candidate genes that may affect both production and fertility traits through intermetabolic network connections 

The SNP BTA-27242-no-rs on BTA5 was significantly associated with PROT and CI. Using a lower threshold for significance (p-value < 5e-04) this SNP was also associated with FRT, ANG and DFS. The SNP had opposite effects on fertility and production traits, in so-far-as the positive allele for PROT had a negative effect on FRT, CI , DFS and ANG. Five genes are located within 1Mbp of this SNP (Table 4), none of which has been extensively studied in cattle or other livestock species. Pathway analysis of the human homologues did not identify functions that could obviously be related to fertility or production traits. 

**Table 4:** Name, Ensembl ID and position of the five genes located within 1Mbp upstream and 1Mbp downstream marker BTA-27242-no-rs. Gene names and positions in human are also indicated.

***Bos taurus* genes**	**Genes**	**Location**	***Homo sapiens* homologues**	**Location**
DUS6_BOVIN	ENSBTAG00000004587	5:19276348-19280028	DUSP6	12:89741050-89747048
A5PKH9_BOVIN	ENSBTAG00000000687	5:19357951-19462573	POC1B	12:89813495-89919801
F1N7J2_BOVIN	ENSBTAG00000009552	5:19539973-19669793	ATP2B1	12:89981828-90103077
CL012_BOVIN	ENSBTAG00000007035	5:20898144-20899948	C12orf12	12:91299399-91348953
EPYC_BOVIN	ENSBTAG00000007990	5:20909663-20950211	EPYC	12:91357456-91398803

 An IPA network analysis of 118 genes located within 1Mbp of the 83 significant SNPs (Table 2) identified 11 networks, 5 of which had high scores. The highest scoring network included 37 proteins, 19 of which were produced by candidate genes near GWAS peaks, 9 for MILK and FAT and 10 for fertility related traits. In this network the interconnection between the 19 genes is mainly mediated by molecules not detected by GWAS. The main functions of the network are cell death, cell signaling and cell development. The same functions involved in cell cycle and in cell death were found also in the 5 other networks identified (4, 5, 9, 10 and 11), however no genes were in common among the networks.

The second network had a score of 34 and contains 18 genes among the 118 close to the significant SNPs (Figure 2). This network includes genes involved in the control of production traits, including *DGAT1* and *APOH* described above, and ATPase, Ca++ transporting plasma membrane 1 (*ATP2B1*) which has been shown to be involved in milk production traits [18,19] and in addition genes having a role in reproduction, including luteinizing hormone (LH) and the follicle-stimulating hormone (FSH) genes. Luteinizing hormone is produced by the anterior pituitary gland, and variations in the concentration of the LH trigger ovulation and development of the *corpus* *luteum* in females, while in males LH is involved in the production of testosterone (Knight PG et al. 2006). LH acts synergistically with FSH to regulate follicle development, embryonic growth, pubertal maturation, and general reproductive processes [24] .

The second network also includes genes connecting LH and FSH with *DGAT1*. These are the platelet-derived growth factor beta polypeptide (*PDGFBB*), transforming growth factor beta 1 (*TGFB1*), and transforming growth factor beta 2 (*TGFB2*) genes. Three genes (*LH*, *FSH* and *PDGFBB*) are known to be involved in ovarian follicle development [24]. In addition the TGFB2 gene is located under the SNP rs41635426 in position 22101902 on BTA16, which was significantly associated with the number of days open (p-value 1.18 e-05), indicating a possible link between fertility and protein content of milk. 

The third network had a score of 30 and includes 16 of the genes close to significant SNP. This network, which includes amino acid metabolism, small molecule signalling and molecular transport, is more closely associated with production traits than with fertility. 

Although the details of the mechanism controlling variation in fertility traits and production traits in cattle still needed to be untangled. The chromosomal regions with significant loci contain genes within pathways described here identify unforeseen relationships that would not be obvious from classical GWA studies and merit further investigation.

## Conclusions

The results presented here support the polygenic nature of the traits analysed. All these traits have low heritability, particularly the fertility traits, which are correlated predictors rather than direct biological measurements. These factors reduce the probability of identifying associations in a genome wide associations in study of this size. Few markers were found in common among the traits: one marker was associated with production (MILK) and ANG on BTA26, and 2 markers on BTA5 were associated with FRT and CI. Only one association, on BTA5, was identified as having effects on both production and fertility traits when a slightly lower significance threshold was applied. 

Marker ARS-BFGL-NGS-4939 on BTA14, which is close to the DGAT1 gene, was found to have an effect on all the production traits analysed. This genetic locus had little or no direct effect on fertility traits, however, network analysis indicated an interconnection between DGAT1 and genes influencing fertility. The complex balance between production and fertility deserves the combination of GWAS and network analysis to provide novel insights into the reproductive biology of dairy cows and the possible connections between fertility and production, justifying the further development of novel and interdisciplinary approaches, as used here.

## Supporting Information

Table S1 **List of Candidate genes liked with fertility obtained from literature review and their relative genomic position.**
(DOCX)

Table S2 **Reference list used to identify functional candidate genes for fertility.**
(DOCX)
