# Supplementary material for: Genome Wide Analysis of Fertility and Production Traits in Italian Holstein Cattle
Source: PLoS One. 2013 Nov 12;8(11):e80219. doi: 10.1371/journal.pone.0080219 (PMC3827211; doi:10.1371/journal.pone.0080219)
Supplement: Table S1 — List of Candidate genes liked with fertility obtained from literature review and their relative genomic position. (DOCX) [file pone.0080219.s001.docx]

**Table S1**

List of Candidate genes liked with fertility obtained from literature review and their relative genomic position.

| Gene name | Start_position | End_position | BTA |
| --- | --- | --- | --- |
| AHSG | 81202132 | 81209114 | 1 |
| BOC | 58428032 | 58464999 | 1 |
| CCDC80 | 57819993 | 57855630 | 1 |
| CD86 | 67144111 | 67210848 | 1 |
| COL18A1 | 146989244 | 147040968 | 1 |
| CP | 119896120 | 119939362 | 1 |
| DNAJC19 | 86666813 | 86672081 | 1 |
| HCLS1 | 66720537 | 66754184 | 1 |
| HES1 | 73973533 | 73976720 | 1 |
| IGSF10 | 117532037 | 117544618 | 1 |
| IL20RB | 133269145 | 133310063 | 1 |
| JAM2 | 10083035 | 10165613 | 1 |
| MCM3AP | 147665788 | 147706423 | 1 |
| MUC13 | 69935163 | 69961881 | 1 |
| MX1 | 143176083 | 143204865 | 1 |
| MX2 | 143119272 | 143155057 | 1 |
| MYLK | 68580144 | 68667822 | 1 |
| PLA1A | 64945861 | 64976251 | 1 |
| PLD1 | 96517509 | 96676250 | 1 |
| POU1F1 | 35009035 | 35024781 | 1 |
| PTX3 | 111027804 | 111033868 | 1 |
| ROBO1 | 25620170 | 26210494 | 1 |
| SELT | 118445768 | 118476097 | 1 |
| TF | 136683311 | 136722309 | 1 |
| USP25 | 20664990 | 20798853 | 1 |
| BBS5 | 26800661 | 26821659 | 2 |
| C1QA | 130792855 | 130795743 | 2 |
| C1QB | 130769172 | 130775911 | 2 |
| C1QC | 130783987 | 130788357 | 2 |
| CD302 | 36656333 | 36681937 | 2 |
| CFLAR | 90139395 | 90172951 | 2 |
| COL3A1 | 7318227 | 7356937 | 2 |
| COL4A4 | 115971118 | 116104632 | 2 |
| CPS1 | 98758036 | 98898492 | 2 |
| DPP4 | 34527583 | 34547390 | 2 |
| EPHA4 | 110251546 | 110405363 | 2 |
| FABP3 | 122723225 | 122783830 | 2 |
| HERC2 | 646582 | 878049 | 2 |
| HSPG2 | 131517579 | 131587498 | 2 |
| IFI6 | 126246561 | 126250182 | 2 |
| IFIH1 | 34217173 | 34272473 | 2 |
| MED18 | 125653307 | 125658882 | 2 |
| MFAP2 | 136187693 | 136192151 | 2 |
| MSTN | 6213566 | 6220196 | 2 |
| NCKAP5 | 65056070 | 65141738 | 2 |
| NMI | 44930495 | 44950481 | 2 |
| NR4A2 | 39999717 | 40017015 | 2 |
| PID1 | 117836412 | 118104185 | 2 |
| SLC35F5 | 65677515 | 65719366 | 2 |
| SMPDL3B | 125983558 | 126015376 | 2 |
| SP140 | 118927759 | 118962018 | 2 |
| STAT1 | 79888206 | 79973705 | 2 |
| SYTL1 | 126519062 | 126526238 | 2 |
| TINAGL1 | 122685823 | 122695535 | 2 |
| TNFAIP6 | 44850892 | 44867293 | 2 |
| TRIP12 | 118622220 | 118729375 | 2 |
| YARS | 121679989 | 121712437 | 2 |
| ZNF142 | 107322277 | 107338174 | 2 |
| AMIGO1 | 34054795 | 34060289 | 3 |
| APCS | 10245766 | 10246803 | 3 |
| APOA2 | 8301476 | 8302729 | 3 |
| ARHGEF11 | 13887504 | 13970875 | 3 |
| BCL2L15 | 29643176 | 29648016 | 3 |
| CADM3 | 10652468 | 10683411 | 3 |
| CKS1B | 15667485 | 15670986 | 3 |
| COL11A1 | 40448699 | 40682012 | 3 |
| DUSP12 | 7849647 | 7857668 | 3 |
| EIF2C3 | 110338488 | 110456098 | 3 |
| FAM46C | 25703050 | 25705109 | 3 |
| FCER1G | 8305544 | 8308776 | 3 |
| FCGR1A | 20847979 | 20856929 | 3 |
| FCGR3A | 7996519 | 8005228 | 3 |
| FHL3 | 108547857 | 108555472 | 3 |
| HAX1 | 16307944 | 16310536 | 3 |
| HECTD3 | 101623083 | 101631050 | 3 |
| HIPK1 | 29512758 | 29563150 | 3 |
| MPZL1 | 930876 | 1012734 | 3 |
| NES | 14208425 | 14215064 | 3 |
| NEXN | 66998944 | 67053966 | 3 |
| PEAR1 | 13986478 | 14007210 | 3 |
| RPS8 | 101816844 | 101818956 | 3 |
| S100A12 | 17163820 | 17165262 | 3 |
| S100A4 | 16887102 | 16888570 | 3 |
| S100A9 | 17176217 | 17179005 | 3 |
| SLC16A1 | 30533845 | 30563298 | 3 |
| SPP2 | 114309328 | 114336917 | 3 |
| SSX2IP | 59590264 | 59648545 | 3 |
| STXBP3 | 34681546 | 34734868 | 3 |
| TACSTD2 | 88032648 | 88033607 | 3 |
| TRIM45 | 26213057 | 26227392 | 3 |
| TSPAN2 | 28364796 | 28449607 | 3 |
| WNT2B | 30942961 | 30957520 | 3 |
| CACNA2D1 | 38712468 | 38856748 | 4 |
| CDK5 | 114429163 | 114433469 | 4 |
| COL1A2 | 11624470 | 11661163 | 4 |
| COPG2 | 95079916 | 95209257 | 4 |
| DNAJB6 | 119431678 | 119447704 | 4 |
| EEPD1 | 61354648 | 61476979 | 4 |
| IL6 | 31578311 | 31582667 | 4 |
| IRF5 | 93706154 | 93717881 | 4 |
| MEST | 95066962 | 95079685 | 4 |
| NOD1 | 66275926 | 66311831 | 4 |
| PDK4 | 12754202 | 12767677 | 4 |
| RELN | 44892394 | 45289293 | 4 |
| REPIN1 | 113602367 | 113604046 | 4 |
| SGCE | 11840470 | 11911742 | 4 |
| STK17A | 78160491 | 78174798 | 4 |
| ABCC9 | 88677586 | 88831324 | 5 |
| ADCY6 | 31160699 | 31176231 | 5 |
| AMIGO2 | 33340318 | 33341883 | 5 |
| BCL2L14 | 98196414 | 98220668 | 5 |
| CKAP4 | 69972287 | 69979795 | 5 |
| ELK3 | 60823240 | 60891359 | 5 |
| FRS2 | 44127711 | 44133546 | 5 |
| GAPDH | 104237902 | 104241979 | 5 |
| GRASP | 28019961 | 28027764 | 5 |
| GTPBP1 | 110846349 | 110868288 | 5 |
| KRT8 | 27213750 | 27221177 | 5 |
| LDHB | 88962679 | 88981219 | 5 |
| LGALS1 | 110014543 | 110017878 | 5 |
| MCM5 | 73990687 | 74009154 | 5 |
| NR4A1 | 27977007 | 27992869 | 5 |
| PAH | 66950501 | 67040705 | 5 |
| PFKM | 32312957 | 32337525 | 5 |
| PLBD1 | 95706361 | 95796211 | 5 |
| SAMM50 | 114915781 | 114953922 | 5 |
| SLC38A2 | 34028553 | 34042996 | 5 |
| SOCS2 | 23523981 | 23528860 | 5 |
| STAT2 | 57318819 | 57333404 | 5 |
| TNFRSF1A | 104402771 | 104415588 | 5 |
| TST | 75810521 | 75817101 | 5 |
| USP18 | 76375512 | 76405820 | 5 |
| WNK1 | 108079510 | 108207083 | 5 |
| ALB | 90232762 | 90251126 | 6 |
| ALPK1 | 14280383 | 14342391 | 6 |
| ARHGAP24 | 102500170 | 102666713 | 6 |
| CXCL10 | 92623587 | 92625937 | 6 |
| CXCL2 | 90695494 | 90697557 | 6 |
| CXCL6 | 90645964 | 90648076 | 6 |
| EMCN | 25578634 | 25700215 | 6 |
| GAK | 108988692 | 109036271 | 6 |
| GC | 88695940 | 88739180 | 6 |
| HSD17B13 | 103971537 | 103991240 | 6 |
| IBSP | 38309555 | 38323305 | 6 |
| IGFBP7 | 74071067 | 74150456 | 6 |
| KDR | 72232849 | 72277699 | 6 |
| KIT | 71796318 | 71917431 | 6 |
| NPNT | 20450525 | 20529959 | 6 |
| SHISA3 | 62877287 | 62880849 | 6 |
| SPON2 | 109224627 | 109228237 | 6 |
| SPP1 | 38120578 | 38127577 | 6 |
| TACR3 | 22642030 | 22730408 | 6 |
| ACP5 | 17134691 | 17137939 | 7 |
| AGXT2L2 | 40644047 | 40669346 | 7 |
| ANGPTL4 | 18236517 | 18243581 | 7 |
| AP3D1 | 22709434 | 22743787 | 7 |
| CD14 | 53446106 | 53448979 | 7 |
| DHFR | 83024158 | 83040758 | 7 |
| DNASE2 | 13790587 | 13792998 | 7 |
| ELANE | 44988172 | 44991299 | 7 |
| FAM125A | 5611162 | 5615225 | 7 |
| FAM32A | 7854536 | 7861328 | 7 |
| GADD45B | 22411968 | 22414079 | 7 |
| GLRX | 97531726 | 97541600 | 7 |
| HBEGF | 53216665 | 53227848 | 7 |
| HSPA4 | 46228919 | 46282857 | 7 |
| INSL3 | 5252672 | 5254252 | 7 |
| IRF1 | 23235653 | 23243697 | 7 |
| MCOLN1 | 17614263 | 17623106 | 7 |
| MLLT1 | 19374861 | 19431741 | 7 |
| PCDHGA8 | 54152475 | 54281944 | 7 |
| PCDHGB4 | 54152475 | 54281944 | 7 |
| PCDHGC3 | 54152475 | 54281944 | 7 |
| PTPRS | 20191965 | 20256722 | 7 |
| SMAD5 | 49155483 | 49217780 | 7 |
| TIMD4 | 70297936 | 70348692 | 7 |
| VCAN | 85666058 | 85782896 | 7 |
| ALDOB | 92773692 | 92865307 | 8 |
| AMBP | 105006306 | 105018465 | 8 |
| ANXA1 | 49624473 | 49642916 | 8 |
| C5 | 112239889 | 112336418 | 8 |
| CTSL1 | 82365269 | 82371037 | 8 |
| DDX58 | 11428373 | 11472719 | 8 |
| DNAJA1 | 76107824 | 76118491 | 8 |
| ELAVL2 | 20374796 | 20452505 | 8 |
| ENTPD4 | 71419941 | 71457015 | 8 |
| FBP1 | 82460864 | 82491694 | 8 |
| GALT | 77352975 | 77356425 | 8 |
| IL6 | 23287349 | 23287909 | 8 |
| IL6 | 23310186 | 23310956 | 8 |
| KIF27 | 78439020 | 78524275 | 8 |
| MSRA | 8635550 | 9019576 | 8 |
| NR4A3 | 65341202 | 65373695 | 8 |
| NTRK2 | 79336288 | 79743284 | 8 |
| OGN | 85453019 | 85468721 | 8 |
| ORM1 | 105216969 | 105220438 | 8 |
| PGM5 | 44682699 | 44892128 | 8 |
| PTPRD | 36575709 | 36797407 | 8 |
| RPS15A | 84843154 | 84843546 | 8 |
| S1PR3 | 90649983 | 90663196 | 8 |
| SCARA5 | 10662268 | 10796378 | 8 |
| SCRG1 | 5632225 | 5644628 | 8 |
| TDH | 7789658 | 7807430 | 8 |
| TEK | 17040335 | 17143857 | 8 |
| TGFBR1 | 64570093 | 64641796 | 8 |
| TPM2 | 60267452 | 60274815 | 8 |
| ARG1 | 70499608 | 70516610 | 9 |
| BRP44L | 102880277 | 102893601 | 9 |
| COL12A1 | 14869511 | 14983763 | 9 |
| ECHDC1 | 24240995 | 24273071 | 9 |
| LAMA4 | 38644310 | 38810902 | 9 |
| PLG | 98016582 | 98064203 | 9 |
| SLC16A10 | 39647514 | 39759908 | 9 |
| SMPDL3A | 28811578 | 28828968 | 9 |
| BBS4 | 19317484 | 19363592 | 10 |
| BHMT | 10127781 | 10151271 | 10 |
| CALM3 | 103059964 | 103067071 | 10 |
| COQ6 | 85725236 | 85740089 | 10 |
| CYP19A1 | 59227895 | 59282939 | 10 |
| DHRS4 | 20922260 | 20934454 | 10 |
| DMGDH | 9994010 | 10066954 | 10 |
| F2RL1 | 7956084 | 7964929 | 10 |
| GALNTL1 | 81396104 | 81494769 | 10 |
| IRF9 | 20820964 | 20825886 | 10 |
| IVD | 36140626 | 36152399 | 10 |
| NPTN | 20207483 | 20270508 | 10 |
| PGF | 86595706 | 86608816 | 10 |
| PTGER2 | 11773706 | 11786080 | 10 |
| RASGRP1 | 34202006 | 34280992 | 10 |
| RNASE6 | 26402507 | 26404000 | 10 |
| SIX1 | 73068706 | 73072802 | 10 |
| TYRO3 | 37078592 | 37095809 | 10 |
| ZFYVE26 | 80180315 | 80245861 | 10 |
| ZWILCH | 13323843 | 13366228 | 10 |
| ABO | 104232297 | 104251043 | 11 |
| ADD2 | 13698135 | 13755248 | 11 |
| CIB4 | 72920571 | 72971981 | 11 |
| COMMD1 | 60427663 | 60598437 | 11 |
| CRIM1 | 18835047 | 19041904 | 11 |
| EFEMP1 | 38338744 | 38408288 | 11 |
| EGFL7 | 104130864 | 104131233 | 11 |
| EIF2AK2 | 19578982 | 19621259 | 11 |
| EIF2AK3 | 47302536 | 47384700 | 11 |
| FABP1 | 47787639 | 47795270 | 11 |
| HK2 | 9723269 | 9766920 | 11 |
| IL1A | 46349422 | 46360402 | 11 |
| IL1B | 46410277 | 46418787 | 11 |
| IL1R2 | 6721485 | 6757777 | 11 |
| IL1RN | 46699166 | 46706152 | 11 |
| LMAN2L | 2653390 | 2672003 | 11 |
| ODF2 | 99047692 | 99074785 | 11 |
| PAPOLG | 43378849 | 43408807 | 11 |
| POLR1A | 48629652 | 48713878 | 11 |
| PTGDS | 106247577 | 106250629 | 11 |
| RPIA | 47220160 | 47254704 | 11 |
| RSAD2 | 90012630 | 90029927 | 11 |
| SLC20A1 | 46218716 | 46232124 | 11 |
| SULT1C3 | 44768842 | 44785035 | 11 |
| TRIB2 | 85205522 | 85233638 | 11 |
| USP20 | 100237005 | 100281194 | 11 |
| CLDN10 | 76758754 | 76864805 | 12 |
| COL4A2 | 89112423 | 89165255 | 12 |
| CPB2 | 16184646 | 16242341 | 12 |
| PAN3 | 31861044 | 31941991 | 12 |
| RPL21 | 32852826 | 32859542 | 12 |
| SHISA2 | 33578134 | 33583714 | 12 |
| SPG20 | 25199123 | 25229866 | 12 |
| TNFSF13B | 87654333 | 87671973 | 12 |
| TNFSF13B | 87679928 | 87684129 | 12 |
| B4GALT5 | 78464294 | 78484987 | 13 |
| BCL2L1 | 61766806 | 61817383 | 13 |
| CD40 | 75563518 | 75574215 | 13 |
| EIF6 | 65206238 | 65212123 | 13 |
| FAM107B | 29501668 | 29578090 | 13 |
| FKBP1A | 60276502 | 60303755 | 13 |
| FLRT3 | 8041873 | 8046999 | 13 |
| GMEB2 | 54573252 | 54583251 | 13 |
| GPR158 | 26229727 | 26526726 | 13 |
| ITIH2 | 16217717 | 16250004 | 13 |
| JAG1 | 3832286 | 3876681 | 13 |
| KIF3B | 62290758 | 62331052 | 13 |
| MRPS26 | 52590818 | 52593105 | 13 |
| MSRB2 | 24392819 | 24417185 | 13 |
| OPTN | 28078762 | 28117627 | 13 |
| PCK1 | 59144594 | 59150719 | 13 |
| PKIG | 73669767 | 73749992 | 13 |
| RIN2 | 39808123 | 39871299 | 13 |
| SEPHS1 | 28288106 | 28314599 | 13 |
| SIGLEC1 | 51945279 | 51962376 | 13 |
| SIRPA | 53667570 | 53710792 | 13 |
| SOX18 | 54295399 | 54296819 | 13 |
| TGM2 | 67663138 | 67697607 | 13 |
| UCKL1 | 54362734 | 54373970 | 13 |
| WISP2 | 73833119 | 73847404 | 13 |
| ASPH | 28700905 | 28888284 | 14 |
| CHRAC1 | 4182522 | 4185763 | 14 |
| COMMD5 | 1531491 | 1533526 | 14 |
| CSPP1 | 33243588 | 33342863 | 14 |
| DERL1 | 18307140 | 18329318 | 14 |
| EIF2C2 | 4085146 | 4168483 | 14 |
| ENPP2 | 83360211 | 83456674 | 14 |
| FABP4 | 46833665 | 46838053 | 14 |
| LY6E | 2624758 | 2628462 | 14 |
| LY96 | 39369425 | 39402263 | 14 |
| MTSS1 | 16971033 | 17132800 | 14 |
| NOV | 47005560 | 47013935 | 14 |
| NRBP2 | 2152612 | 2159648 | 14 |
| SDC2 | 69930853 | 70050653 | 14 |
| VCPIP1 | 32884899 | 32908633 | 14 |
| AASDHPPT | 1778699 | 1802177 | 15 |
| APBB1 | 47109758 | 47267567 | 15 |
| APOA4 | 27906599 | 27909370 | 15 |
| APOC3 | 27914680 | 27917264 | 15 |
| ARFIP2 | 47186494 | 47191611 | 15 |
| ARRB1 | 55255745 | 55329451 | 15 |
| CASP4 | 3474706 | 3494168 | 15 |
| CD44 | 66454331 | 66541790 | 15 |
| CRYAB | 22566929 | 22570256 | 15 |
| DCHS1 | 47042652 | 47064122 | 15 |
| DLAT | 22665193 | 22697952 | 15 |
| HMBS | 30194980 | 30202275 | 15 |
| HPX | 47226087 | 47235666 | 15 |
| IL18BP | 52416834 | 52418796 | 15 |
| LRP4 | 77663792 | 77701236 | 15 |
| MCAM | 30414474 | 30422213 | 15 |
| MICAL2 | 41003243 | 41107850 | 15 |
| MRPL48 | 54065466 | 54120351 | 15 |
| NLRX1 | 30278100 | 30296544 | 15 |
| SERPING1 | 82159474 | 82172143 | 15 |
| SPON1 | 38991044 | 39309268 | 15 |
| TCN1 | 84389636 | 84404937 | 15 |
| AGRN | 52674207 | 52712338 | 16 |
| BTG2 | 890072 | 892370 | 16 |
| CD55 | 5105577 | 5124229 | 16 |
| CFH | 6052925 | 6122550 | 16 |
| ELK4 | 3208946 | 3219936 | 16 |
| FMOD | 925081 | 935131 | 16 |
| GLUL | 64948332 | 64958885 | 16 |
| HMCN1 | 68192901 | 68733392 | 16 |
| IL10 | 4402474 | 4406421 | 16 |
| IRF6 | 75401221 | 75417466 | 16 |
| ISG15 | 52714627 | 52715665 | 16 |
| MXRA8 | 52424102 | 52428456 | 16 |
| MYOC | 39957137 | 39971282 | 16 |
| NCF2 | 66081122 | 66128113 | 16 |
| PIGR | 4533505 | 4551569 | 16 |
| PRELP | 1041923 | 1055957 | 16 |
| SELL | 38158186 | 38173226 | 16 |
| SELP | 38049259 | 38080862 | 16 |
| SOAT1 | 62068139 | 62128359 | 16 |
| TGFB2 | 22495736 | 22588280 | 16 |
| VWA1 | 52360276 | 52365505 | 16 |
| ATP2A2 | 56458750 | 56512895 | 17 |
| CLDN5 | 74749250 | 74750524 | 17 |
| EDNRA | 10752466 | 10828318 | 17 |
| EIF4ENIF1 | 72293386 | 72332007 | 17 |
| FGA | 2842939 | 2849702 | 17 |
| FGB | 2875550 | 2884552 | 17 |
| FGG | 2823021 | 2830960 | 17 |
| IGLL1 | 72826680 | 73152862 | 17 |
| KCTD10 | 65950166 | 65979498 | 17 |
| MMP11 | 73216822 | 73220883 | 17 |
| SFRP2 | 3829564 | 3838136 | 17 |
| SMPD4 | 74397215 | 74412807 | 17 |
| TDO2 | 44377634 | 44394268 | 17 |
| TRIM2 | 4308229 | 4436083 | 17 |
| APOC2 | 53057717 | 53059957 | 18 |
| BCL2L12 | 56523439 | 56530499 | 18 |
| CALM3 | 54169313 | 54178928 | 18 |
| CDH1 | 36221702 | 36243479 | 18 |
| CDH11 | 32798826 | 32888927 | 18 |
| CDH13 | 9512739 | 10162782 | 18 |
| CES1 | 24031967 | 24058134 | 18 |
| CES1 | 24066409 | 24098654 | 18 |
| CIRH1A | 36471594 | 36500063 | 18 |
| CRISPLD2 | 10985132 | 11050904 | 18 |
| DDX19B | 1799804 | 1821405 | 18 |
| FCGRT | 56415700 | 56421861 | 18 |
| HP | 39173135 | 39179293 | 18 |
| IRF3 | 56517723 | 56523302 | 18 |
| IRF8 | 11883376 | 11905043 | 18 |
| MIA | 50312668 | 50314099 | 18 |
| MT3 | 24134095 | 24135544 | 18 |
| PSENEN | 46646707 | 46648031 | 18 |
| RPS15A | 39393580 | 39393972 | 18 |
| SIGLEC10 | 57835594 | 57842767 | 18 |
| SIGLEC14 | 57971424 | 57975412 | 18 |
| SIGLEC14 | 57997837 | 58000711 | 18 |
| SPHK2 | 55714370 | 55721629 | 18 |
| SULT2A1 | 55173175 | 55191604 | 18 |
| TUBB6 | 14761260 | 14769914 | 18 |
| AATK | 52181458 | 52196943 | 19 |
| ALOX12 | 27421125 | 27434258 | 19 |
| ALOX15 | 27331075 | 27339653 | 19 |
| APOH | 63230261 | 63243064 | 19 |
| CA4 | 13109655 | 13117426 | 19 |
| CCL11 | 16196626 | 16199525 | 19 |
| CCL16 | 14782701 | 14786690 | 19 |
| CCL2 | 16232968 | 16234839 | 19 |
| CCL8 | 16137674 | 16139707 | 19 |
| CCR7 | 41428917 | 41440070 | 19 |
| CD300LG | 44356959 | 44368596 | 19 |
| CDC42EP4 | 58620114 | 58622767 | 19 |
| COL1A1 | 37088246 | 37104998 | 19 |
| EVI2B | 19027998 | 19036601 | 19 |
| HEXDC | 50772368 | 50788334 | 19 |
| HEXIM2 | 45452267 | 45459216 | 19 |
| HSD17B1 | 43268326 | 43270819 | 19 |
| KRT10 | 41663605 | 41667960 | 19 |
| LGALS3BP | 54035259 | 54045168 | 19 |
| MED11 | 27253471 | 27255303 | 19 |
| MFAP4 | 34685357 | 34687892 | 19 |
| MRC2 | 47721711 | 47747246 | 19 |
| NOS2 | 19758764 | 19956549 | 19 |
| PECAM1 | 49175892 | 49238414 | 19 |
| PEMT | 35389675 | 35420462 | 19 |
| SLC16A3 | 51243972 | 51255385 | 19 |
| SPHK1 | 55978951 | 55981068 | 19 |
| STAT5A | 43033597 | 43054075 | 19 |
| SUPT4H1 | 9540309 | 9546074 | 19 |
| VTN | 20413683 | 20416614 | 19 |
| XAF1 | 25759759 | 25772925 | 19 |
| YWHAE | 23086308 | 23121103 | 19 |
| EGFLAM | 35999051 | 36178472 | 20 |
| FST | 25588642 | 25594057 | 20 |
| GHR | 31890736 | 32199996 | 20 |
| HEXB | 6721327 | 6753670 | 20 |
| MAP1B | 9330175 | 9419040 | 20 |
| OSMR | 35521410 | 35588186 | 20 |
| PRLR | 39073246 | 39137480 | 20 |
| PTGER4 | 33762479 | 33774648 | 20 |
| SEMA5A | 64147933 | 64352311 | 20 |
| SNX18 | 24570227 | 24596518 | 20 |
| ATXN3 | 57347865 | 57381142 | 21 |
| CLEC14A | 48843729 | 48845622 | 21 |
| COMMD4 | 33872674 | 33878166 | 21 |
| FBLN5 | 57153110 | 57246389 | 21 |
| ISLR | 34903758 | 34906887 | 21 |
| JAG2 | 71145150 | 71156637 | 21 |
| LOXL1 | 35057678 | 35082119 | 21 |
| LRFN5 | 51772760 | 52095083 | 21 |
| LRRK1 | 5642546 | 5734611 | 21 |
| MAP1A | 55810061 | 55829462 | 21 |
| SERPINA1 | 59578528 | 59587897 | 21 |
| SERPINA11 | 59650976 | 59664635 | 21 |
| SERPINA3 | 60956931 | 60986261 | 21 |
| SERPINA5 | 59782814 | 59795808 | 21 |
| SIVA1 | 70870027 | 70874415 | 21 |
| SLC28A1 | 22949800 | 23048041 | 21 |
| ARHGEF3 | 44810834 | 44880156 | 22 |
| ATG7 | 55944219 | 56217921 | 22 |
| CRTAP | 7532626 | 7565025 | 22 |
| CSPG5 | 52649429 | 52661597 | 22 |
| DNASE1L3 | 43639336 | 43658740 | 22 |
| EIF4E3 | 30019806 | 30078465 | 22 |
| ITIH1 | 48655027 | 48669271 | 22 |
| MOBP | 12767735 | 12799652 | 22 |
| MST1R | 50928327 | 50941427 | 22 |
| NICN1 | 51262430 | 51266667 | 22 |
| PRKCD | 48307244 | 48337780 | 22 |
| RBM15B | 49941346 | 49944165 | 22 |
| SHISA5 | 51944064 | 51969644 | 22 |
| STAB1 | 48884238 | 48909562 | 22 |
| TIMP4 | 57602210 | 57609085 | 22 |
| TREX1 | 51969881 | 51971755 | 22 |
| AIF1 | 27503183 | 27505457 | 23 |
| BPHL | 50406551 | 50408215 | 23 |
| C2 | 27231725 | 27243367 | 23 |
| CD83 | 42335199 | 42354922 | 23 |
| CFB | 27225270 | 27231292 | 23 |
| CYP39A1 | 19776642 | 19860368 | 23 |
| DAXX | 7429817 | 7434186 | 23 |
| FGD2 | 10892083 | 10918349 | 23 |
| FKBP5 | 9521254 | 9637802 | 23 |
| FLOT1 | 28090029 | 28100113 | 23 |
| GMDS | 51117008 | 51453402 | 23 |
| GNMT | 16579606 | 16583028 | 23 |
| GPLD1 | 32984456 | 33036038 | 23 |
| GSTA2 | 24823643 | 24837430 | 23 |
| KLHDC3 | 16627392 | 16638402 | 23 |
| LY6G6C | 27407611 | 27410285 | 23 |
| NFKBIL1 | 27550617 | 27560647 | 23 |
| NOTCH4 | 26973191 | 26998620 | 23 |
| PRL | 35105135 | 35113759 | 23 |
| RGL2 | 7407899 | 7414957 | 23 |
| TAP1 | 7117863 | 7126822 | 23 |
| TNF | 27533911 | 27536674 | 23 |
| TNXB | 27085415 | 27136954 | 23 |
| TUBB | 28101727 | 28105320 | 23 |
| WRNIP1 | 50703507 | 50714364 | 23 |
| AQP4 | 30362738 | 30377272 | 24 |
| CDH2 | 28992666 | 29241119 | 24 |
| GALNT1 | 21687846 | 21756844 | 24 |
| MEP1B | 25292754 | 25328875 | 24 |
| STARD6 | 54186472 | 54203204 | 24 |
| TTR | 25854675 | 25863535 | 24 |
| ARHGAP17 | 22843459 | 22900615 | 25 |
| ASL | 28247949 | 28256721 | 25 |
| CDR2 | 20218558 | 20247294 | 25 |
| LITAF | 10288891 | 10325021 | 25 |
| MGRN1 | 3754935 | 3791613 | 25 |
| NDUFB10 | 1517626 | 1520287 | 25 |
| NUPR1 | 26340079 | 26341408 | 25 |
| PDIA2 | 309841 | 312520 | 25 |
| PRSS22 | 2179213 | 2183525 | 25 |
| PRSS8 | 27485086 | 27489416 | 25 |
| PYCARD | 27541409 | 27542740 | 25 |
| RPS15A | 16531761 | 16537583 | 25 |
| SOCS1 | 9975299 | 9975970 | 25 |
| TGFB1I1 | 27760811 | 27766954 | 25 |
| ACTA2 | 10662362 | 10714982 | 26 |
| BAG3 | 40118556 | 40141989 | 26 |
| CPXM2 | 43829293 | 43966907 | 26 |
| CYP2C19 | 16336198 | 16383496 | 26 |
| CYP2E1 | 50550575 | 50561259 | 26 |
| DKK1 | 6852970 | 6855647 | 26 |
| EXOSC1 | 18522299 | 18528929 | 26 |
| FGFR2 | 41823653 | 41926635 | 26 |
| RBP4 | 14940551 | 14946750 | 26 |
| SFXN3 | 21731746 | 21738756 | 26 |
| TACC2 | 42323743 | 42485654 | 26 |
| ASAH1 | 18277522 | 18309256 | 27 |
| FGL1 | 18454362 | 18472436 | 27 |
| MCPH1 | 4313369 | 4554747 | 27 |
| MYOM2 | 312538 | 376193 | 27 |
| PCM1 | 18328847 | 18405603 | 27 |
| COL13A1 | 26253844 | 26406118 | 28 |
| SUPV3L1 | 25712751 | 25737804 | 28 |
| TSPAN15 | 25960051 | 26012583 | 28 |
| TUBB | 3159664 | 3160998 | 28 |
| APLP2 | 36878509 | 36906465 | 29 |
| ARL2 | 43845378 | 43853177 | 29 |
| ATL3 | 42617154 | 42658661 | 29 |
| CDKN1C | 49368787 | 49370785 | 29 |
| DHCR7 | 48930324 | 48950644 | 29 |
| FEZ1 | 29274999 | 29317604 | 29 |
| GSTP1 | 46087142 | 46090005 | 29 |
| IRF7 | 50903719 | 50906820 | 29 |
| ROBO4 | 28719989 | 28734705 | 29 |
| RSF1 | 18284512 | 18426104 | 29 |
| SIAE | 28485872 | 28523643 | 29 |
| STX5 | 41809627 | 41837545 | 29 |
| TALDO1 | 50856122 | 50863486 | 29 |
| WDR74 | 41838794 | 41846745 | 29 |
| AIFM1 | 13966656 | 13997438 | X |
| CA5B | 135022449 | 135049835 | X |
| CFP | 91441704 | 91447087 | X |
| CITED1 | 83495862 | 83501893 | X |
| COL4A5 | 61685327 | 61824307 | X |
| EMD | 40341311 | 40343613 | X |
| F9 | 23025837 | 23059616 | X |
| FLNA | 40310757 | 40332714 | X |
| LAMP2 | 4846778 | 4879916 | X |
| LONRF3 | 3237205 | 3272604 | X |
| MID1IP1 | 109949360 | 109951138 | X |
| MST4 | 15811930 | 15878198 | X |
| NLGN3 | 84765209 | 84787720 | X |
| TSPAN6 | 50904005 | 50912844 | X |
|  |  |  |  |
