# Supplementary material for: Genome Wide Analysis of Fertility and Production Traits in Italian Holstein Cattle
Source: PLoS One. 2013 Nov 12;8(11):e80219. doi: 10.1371/journal.pone.0080219 (PMC3827211; doi:10.1371/journal.pone.0080219)
Supplement: Table S2 — Reference list used to identify functional candidate genes for fertility. (DOCX) [file pone.0080219.s002.docx]

| 1 | J Dairy Sci. 2010 May;93(5):2244-9. doi: 10.3168/jds.2009-2805. Short communication: Validation of in vitro fertility genes in a Holstein bull population. Khatib H, Monson RL, Huang W, Khatib R, Schutzkus V, Khateeb H, Parrish JJ. |
| --- | --- |
| 2 | J Dairy Sci. 2009 May;92(5):2238-47. doi: 10.3168/jds.2008-1767.Single gene and gene interaction effects on fertilization and embryonic survival rates in cattle. Khatib H, Huang W, Wang X, Tran AH, Bindrim AB, Schutzkus V, Monson RL, Yandell BS. |
| 3 | J Dairy Sci. 2012 Oct;95(10):5657-75. doi: 10.3168/jds.2011-5114. Epub 2012 Aug 9. Effects of lactation and pregnancy on gene expression of endometrium of Holstein cows at day 17 of the estrous cycle or pregnancy. Cerri RL, Thompson IM, Kim IH, Ealy AD, Hansen PJ, Staples CR, Li JL, Santos JE, Thatcher WW. |
| 4 | Reprod Biol Endocrinol. 2009 May 29;7:55. doi: 10.1186/1477-7827-7-55. Expression of genes associated with immunity in the endometrium of cattle with disparate postpartum uterine disease and fertility. Herath S, Lilly ST, Santos NR, Gilbert RO, Goetze L, Bryant CE, White JO, Cronin J, Sheldon IM. |
| 5 | Biology of Reproduction 84, 299–307 (2011) Published online before print 6 October 2010.DOI 10.1095/biolreprod.110.085589. Differential Gene Expression in Ovaries of Pregnant Pigs with High and LowProlificacy Levels and Identification of Candidate Genes for Litter Size. Amanda Fernandez-Rodriguez,Maria Munoz, Almudena Fernandez, Ramona N.Pena, Anna Tomas, Jose L. Noguera, Cristina Ovilo, and Ana I. Fernandez. |

**Table S2**

Reference list used to identify functional candidate genes for fertility.
